# Assessment of BED HIV-1 Incidence Assay in Seroconverter Cohorts: Effect of Individuals with Long-Term Infection and Importance of Stable Incidence

**DOI:** 10.1371/journal.pone.0014748

**Published:** 2011-03-04

**Authors:** Janet M. McNicholl, J. Steven McDougal, Punneeporn Wasinrapee, Bernard M. Branson, Michael Martin, Jordan W. Tappero, Philip A. Mock, Timothy A. Green, Dale J. Hu, Bharat Parekh, for the Thai-U.S. BED Assay Validation Working Group

**Affiliations:** 1 Division of HIV/AIDS Prevention, Centers for Disease Control and Prevention, Atlanta, Georgia, United States of America; 2 The Thailand Ministry of Public Health - U.S. Centers for Disease Control and Prevention Collaboration, Nonthaburi, Thailand; University of Cape Town, South Africa

## Abstract

**Background:**

Performance of the BED assay in estimating HIV-1 incidence has previously been evaluated by using longitudinal specimens from persons with incident HIV infections, but questions remain about its accuracy. We sought to assess its performance in three longitudinal cohorts from Thailand where HIV-1 CRF01_AE and subtype B′ dominate the epidemic.

**Design:**

BED testing was conducted in two longitudinal cohorts with only incident infections (a military conscript cohort and an injection drug user cohort) and in one longitudinal cohort (an HIV-1 vaccine efficacy trial cohort) that also included long-term infections.

**Methods:**

Incidence estimates were generated conventionally (based on the number of annual serocoversions) and by using BED test results in the three cohorts. Adjusted incidence was calculated where appropriate.

**Results:**

For each longitudinal cohort the BED incidence estimates and the conventional incidence estimates were similar when only newly infected persons were tested, whether infected with CRF01_AE or subtype B′. When the analysis included persons with long-term infections (to mimic a true cross-sectional cohort), BED incidence estimates were higher, although not significantly, than the conventional incidence estimates. After adjustment, the BED incidence estimates were closer to the conventional incidence estimates. When the conventional incidence varied over time, as in the early phase of the injection drug user cohort, the difference between the two estimates increased, but not significantly.

**Conclusions:**

Evaluation of the performance of incidence assays requires the inclusion of a substantial number of cohort-derived specimens from individuals with long-term HIV infection and, ideally, the use of cohorts in which incidence remained stable. Appropriate adjustments of the BED incidence estimates generate estimates similar to those generated conventionally.

## Introduction

The development of serologic assays to detect recent HIV-1 infection and to estimate HIV-1 incidence has generated widespread interest in applying this approach to monitor the HIV epidemic [1]–[4] and to identify appropriate populations for efficacy trials. Although incidence has previously been estimated from serial prevalence data and survival assumptions, back-calculation from AIDS case reporting, self-reported serologic history, or passive anonymous/linked surveys [5]–[7], [9]–[11], the accurate estimation of incidence has traditionally relied on prospective HIV-1 testing and longitudinal follow-up of people at risk [12]–[15]. However, these cohort studies are time consuming, logistically difficult, expensive, and subject to biases related to enrollment, behavior change, preventive measures, interventions, loss to follow-up and other study effects. Laboratory assays to determine incidence by using cross-sectional sampling could obviate some of these problems.

In the last decade, a number of assays have been described for the detection of recent HIV-1 infections (reviewed in [3], [4]). These assays rely on features of early HIV-1 infection such as the presence of virus before antibody seroconversion (HIV-1 RNA or HIV-1 p24 antigenemia) or the characteristics of antibody titer, proportion, specificity, isotype, or avidity that differ between early and established infection. These assays define the duration of a transient state related to the evolving response to HIV-1 infection. The prevalence of this transient state in the at-risk population divided by its duration is an estimate of incidence (new infections per person per unit of time).

Before application to population surveys, the assay-defined demarcation of recent from longer-term infection and the mean period of time that the recent state persists in the normal evolution of infection (the recency period of the assay) are determined using specimens collected serially from recently infected people (seroconversion panels). Assay parameters developed and defined in this way are predicted to apply to cross-sectional population samples, although there may be subtle or unforeseen reasons why they do not. For instance, the recency period for a given assay may differ by HIV-1 subtype [16], requiring selection of the appropriate recency period for populations in areas such as Thailand, where more than one subtype predominates. Another factor is the prevalence of false-recent infections. The BED estimates can be adjusted to account for the proportion of false-recent infections [17]–[19].

In this study, we evaluated an assay for recent infection, the BED capture enzyme immunoassay (BED-CEIA, abbreviated as BED in this manuscript) [20], [21] . The BED assay measures the proportion of IgG that is directed against the immunodominant region of HIV-1 gp41. The target antigen is a branched peptide containing consensus gp41 sequences from multiple HIV-1 subtypes [21]. Responses to this peptide rise during the first 2 years following seroconversion, as measured by the BED [16], [21]. The BED assay was applied to specimens from three cohort studies in Thailand, where CRF01_AE and subtype B′ account for the majority of HIV-1 infections. The cohort studies enrolled seronegative participants in three cohorts at different times and places, with different risks, with conventionally estimated incidences varying from 1.2 to 7.0 per 100 person-years (PY) and with varying durations of follow-up. In one cohort, the proportion of persons with long-term infections increased over 3 years, allowing evaluation of the impact of long-term infections. The conventionally estimated incidences were compared to the BED-estimated incidences before and after adjustment.

## Materials and Methods

### Cohorts

#### The Royal Thai Army conscripts study

This study was conducted in Northern Thailand during 1991–1993 [12], [22]. HIV-1 seronegative male conscripts (n = 1115) entering the military were screened for HIV-1 seroconversion at 5, 17, and 23 months. Adherence to follow-up was excellent, and all seropositive specimens were available for BED testing.

#### The Bangkok Metropolitan Administration (BMA) study

This study was conducted in 15 BMA narcotic treatment clinics during 1995–1998 [13], [23]. Injection drug users (IDUs) (n = 1209) without serologic evidence of HIV-1 infection were enrolled from mid 1995 through 1996 and tested for HIV approximately every 4 months for >2 years. Cross-sectional analyses were performed on specimens collected at 8, 16, and 24 months after enrollment and at the end of each calendar year. Specimens from 91 of 120 seroconverters were available for testing. Molecular subtyping of HIV-1 was performed as described elsewhere [24].

#### The AIDSVAX B/E/phase 3 HIV-1 vaccine trial (Vax003)

This was a randomized, double-blind, placebo-controlled, efficacy trial of AIDSVAX B/E, a bivalent recombinant gp120 vaccine (VaxGen, Inc., Brisbane, California) [25] known not to induce immune responses to the region of gp41 used in the BED assay [21], [26]. The trial was conducted in 17 BMA narcotic treatment clinics from March 1999–June 2003. HIV-1–seronegative participants (n = 2545) were tested for serologic evidence of HIV-1 infection every 6 months for 36 months (90% completed follow-up). Seroconverters were retested within a few weeks of their first seropositive test and followed up every 4 months thereafter. Banked specimens collected between May 1 and August 31 of years 2000, 2001, and 2002 represented the cross-sectional populations. Specimens were available for most (193) seroconverters. No one contributed more than one specimen for each collection period.

#### Ethical review

All participants gave written informed consent for HIV testing. The studies were approved by ethical committees of the Centers for Disease Control and Prevention (CDC), the Ministry of Public Health, and the BMA. The CDC IRB numbers were 1825, 2076, 2255. The details of each study can be provided by J. McNicholl or P. Wasinrapee, including consent forms for HIV testing.

#### BED capture enzyme immunoassay

The BED was performed on HIV-1–seropositive specimens as previously described [21]. An 0.8 cutoff value for the normalized optical density (ODn) was used to demarcate “recent” from “established” infection status. Using BED data from 190 seroconversion panels from Thailand [16], we determined the mean period from initial seroconversion to an ODn of 0.8 (the recency period) as 152 days.

#### Comparison of conventionally and BED-estimated incidences

Conventionally, incidence in cohorts is estimated by the number of new infections per 100 PY of observation. These data were available for the three cohorts. To estimate incidence by the BED assay, seropositive specimens from these studies were tested for recent infection. The number of seropositive specimens that were classified as “recent” by the BED assay (N_inc_) divided by the recency period in days (*w*) gave the number of incident infections per day. This number times the number of days in one year (365) gave the annualized BED-estimated number of incident infections: ([N_inc_/*w*]×365). The relevant comparison for this study is the conventionally estimated number of incident infections during a given period versus the BED-estimated number of incident infections for the same period (i.e. the numerators in the incidence formulae). The BED cross-sectional estimation necessarily uses an “at risk” formula: BED-estimated recent infections in one year per number of persons at risk. The number at risk is the number of seronegative persons plus the estimated number of recently infected persons in the screened population. Since the statistical treatment and results are the same with either denominator, we arbitrarily present only the PY-derived incidence values in the tables. Binomial 95% confidence intervals (CIs) were calculated for conventionally and BED-estimated incidences by using a calculation worksheet provided by A.Welte [17]. Differences between conventionally and BED-estimated incidences were considered insignificant (p>0.05) if the 95% confidence intervals of the estimates overlapped.

When specimens from all the seroconverters were not available for testing, the BED estimate was extrapolated by the factor (No. of seroconverters/No. of seroconverter specimens available for testing). This assumes that the proportion of specimens that test positive for recent infection is the same for persons with available specimens and for those whose specimens were missing. The calculation of confidence and the statistical comparisons are based on data from tested specimens, not from extrapolated data. In analyses of long-term specimens (defined as specimens from seroconverters infected in prior years), data were adjusted for misclassification by using a recently described formula [17].

## Results

In the Royal Thai Army conscripts study (Table 1), men were enrolled at the same time and followed up regularly. There were 14 seroconverters in this study. There was general agreement between the conventionally estimated and BED-estimated incidence for all periods. The overall difference in BED-estimated (1.43 per 100 PY) and conventionally estimated (1.19 per 100 PY) incidence was 0.24 per 100 PY, which is not significant.

**Table 1:** Comparison of conventionally and BED-estimated incidence in two cohorts.

Time Period	PY	No. at risk	Estimated No. of incident infections		Annualized incidence/100 PY	
			Conventional	BED	Conventional (CI)	BED (CI)
**Thailand military conscript cohort**						
Months						
5	324	867	2	2.0	0.62 (0.00–1.48)	1.49 (0.00–3.57)
17	660	662	9	9.5	1.38 (0.47–2.29)	1.48 (0.02–2.93)
23	207	472	3	1.2	1.47 (0.00–3.15)	1.18 (0.00–3.49)
Subtotal	1191		14	12.7	1.19 (0.56–1.82)	1.43(0.36–2.49)
**BMA injection drug user cohort**						
Months						
8	709	942	52	34	7.91 (5.68–10.15)	5.22 (4.65–9.87)
16	620	805	38	34	6.53 (4.39–8.67)	5.85 (0.48–11.22)
24	515	668	30	28	6.19 (3.90–8.47)	5.77 (0.00–14.43)
Subtotal	1844		120	96	6.96 (5.67–8.25)	5.49 (2.22–8.76)

PY, person-years; CI, 95% confidence interval; BMA, Bangkok Metropolitan authority.

In the BMA study (Table 1), the annualized conventionally estimated incidence was 6.96 per 100 PY, and the BED-estimated incidence was 5.49 per 100 PY. This overall difference of 1.47 per 100 PY was not significant. With the exception of the analysis for the first 8 months of enrollment, the two incidence estimates were not significantly different for the individual periods. For the first 8 months of enrollment, the BED-estimated incidence (5.22/100 PY) was lower than the conventionally estimated incidence (7.91/100 PY). Conventionally estimated incidence was not stable during the first 8 months. More seroconversions occurred during the first 4 months of enrollment than during the second 4 months, and the annual conventionally estimated incidences, calculated separately for the first and second 4-month periods, were 9.49/100 PY and 5.00/100 PY, respectively. The BED estimate is determined by the number of seroconverters who are within the recency period at the time of specimen collection. Thus, the BED incidence estimate, based on specimens collected at the end of the 8-month interval, corresponds more closely to the conventional estimate for the second 4 months than it does to the conventional estimate for the first 4 months or the first 8 months. We could not analyze consecutive 4-month periods because the sample frame overlapped with the recency period, leading to possible truncation of the BED estimate.

In the BMA study, we compared incidence measures separately for infection with HIV-1 CRF01_AE and subtype B′, the predominant subtypes in Thailand. For CRF01_AE, the conventionally estimated incidence was 5.37 per 100 PY (95% CI, 4.26–6.49), and the BED estimate was 3.91 per 100 PY (95% CI, 1.20–6.62). For subtype B′, the respective measures were 1.43 (95% CI, 0.88–1.98) and 1.43 (95% CI, 0.00–3.02).

In the Vax003 trial (Table 2) we were able to examine the effect of long-term infections. There were 203 seroconverters. Overall, BED-estimated (3.73 per 100 P-Y) and conventionally estimated incidences (3. 71 per 100 P-Y) did not differ significantly. Comparison of all the annualized incidence results in the column labeled “conventional” incidence and the first column (without LT) in the section labeled “BED” shows that the data are similar. These analyses were confined to specimens from participants who had seroconverted and been tested in the indicated year. As the study progressed, specimens collected in the designated year from participants who had seroconverted in previous years became available. As data from these long-term participants accumulated, there was inflation of the BED estimate (compare BED column “without LT” with column “with LT”). For example, when long-term specimens were added in the year 2002, the BED estimate increased from 4.38 to 6.03. When these data were adjusted for misclassification, by using a recently described formula [17], the adjusted BED estimates were lower. The overall adjusted BED estimate was 3.75 per 100 PY, much closer to the conventionally estimated incidence of 3.71 per 100 PY (see Table 2, column labeled “with LT ” compared to column labeled “ with LT (adjusted)” and to column labeled “conventional”). This comparison indicates the importance of adjusting the BED estimate when the analysis includes persons with long-term infections, as would be typical in most cross-sectional surveys.

**Table 2:** Comparison of conventionally and BED-estimated incidence: effect of long term infections.

Test Year	PY	No. at risk	Estimated No. of incident infections			Annualized incidence/100 PY			
			Conventional	BED		Conventional (CI)	BED (CI)		
				WithoutLT	With LT		WithoutLT	WithLT	WithLT (adjusted)
2000	1551	2469	52	69	N/A	3.47 (2.51–4.43)	4.65 (2.64–6.66)	N/A	N/A
2001	2295	2347	71	58	70	3.19 (2.44–3.95)	2.64 (1.49–3.78)	3.17 (1.94–4.39)	2.59 (1.37–3.82)
2002	1836	1737	80	76	107	4.56 (3.53–5.58)	4.38 (2.47–6.28)	6.03 (4.00–8.06)	4.86 (2.84–6.88)
Total	5682		203	203	246	3.71 (3.19–4.22)	3.73 (2.78–4.68)	4.41 (3.43–5.39)	3.75 (2.77–4.73)

PY, person-years; CI, 95% confidence interval; LT, long-term infections. Adjustments were done as recommended by Welte et al. 2009 [reference 17].

## Discussion

In these studies, BED testing was performed on specimens from the same cohorts where incidence was conventionally estimated. The use of the same specimens is an advantage over study designs where BED-estimated incidence is determined on specimens that are related to, but separate from, those of the referent cohort. For instance, the true incidence in the pre-enrollment screening for a cohort study or in a separate cross-section of the same population may not be equivalent to the incidence measured in the related cohort because of selection bias, recruitment bias, or both. The design also allows evaluation of the window and allows subanalysis of features that affect assay performance. We were alerted to two such features in this study: the effect of unstable incidence and the influence of specimens from long-term infected participants.

Incidence estimates from cohorts and from cross-sectional analysis differ fundamentally. Cohort data are collected during prescribed periods, whereas the cross-sectional method produces an estimate at a given point in time. Cohort data measure the number of seroconversions that occur during a given period of follow-up and are frequently used as the criterion standard. The cross-sectional analysis is dependent on the number of seroconverters who are within the recency period (in this instance, 152 days) at the time of specimen collection. The incidence rate that is actually measured is the number of recent infections per 152 days. Extrapolation of this value to a longer period (e.g., 365 days for an annualized estimate) is based on the assumption that the rate remains the same. If the rate is not constant, the BED estimate, extrapolated to a period longer than the recency period, will be in error. An example of this occurred during the first 8 months of follow-up in the BMA study, when more seroconversions during the early part of the period biased the BED estimate, resulting in an underestimate of the conventionally estimated incidence (Table 1). This observation highlights the importance of understanding the relationship of recency period to the sampling period of a cohort study and how fluctuations in incidence during the sampling frame may bias BED results.

The recency period used for calculating BED incidence in this study was 152 days. This period was based on analysis of seroconversion panels from 190 seroconverters representing subtypes B′ and CRF01_AE [16]. The recency period that would have given precise agreement between conventionally and BED-estimated incidences can be calculated by entering the conventionally estimated incidence into the BED incidence formula and solving for the recency period. This calculation would result in a recency period of 140 days (range, 130–153 days). Thus, the recency period used and the recency period that would have given perfect concordance in the conventionally estimated and BED-estimated incidences are similar.

In the Vax003 cohort, the effect of long-term infections was observed. The BED estimate was based on analysis of specimens collected at the end of each year from those who seroconverted in that year (Table 2). As the study progressed, specimens collected in the designated year from participants who had seroconverted in previous years (longer-term infected persons) became available. These specimens should, in theory, be classified as long-term infections in the BED assay, but approximately 5% of these specimens register false-recent BED results [18], [19]. As more and more specimens from participants infected for longer periods of time accumulated, the prevalence of seropositive persons in the cross-sectional samples rose. Consequently, the small portion of false-recent BED results inflated the BED-estimated incidence (Table 2). This analysis is most relevant to the context in which the BED assay is currently widely used: cross-sectional populations with a predominance of longer-term infected people.

The inflation of the incidence estimate related to false-recent results for long-term infected subjects can be substantial, rises with increased prevalence in the test population, and has been noted before [17]–[19], [21], [27]–[29]. The need for further studies in this regard has been pointed out by many [18]–[20], [27], [30], [31]. There are several potential ways of mitigating these effects. To some extent, persons who are known to have long-standing infection can be classified as having long-term infection as part of case-based surveillance [31]. Participants who self-report or otherwise are known to be long-term HIV-1–seropositive [6], [8], patients with AIDS [11], [15], or patients receiving antiretroviral therapy [31], [32] are unlikely to be recently infected and likely to register recent by the assay. This history may be available or can be included in the design of the cross-sectional study and can complement the testing classification. A more stringent testing algorithm could be used, one that requires confirmation of BED-recent specimens with a second test for recent infection and the addition of testing for the presence of antiretroviral drugs in specimens that are BED-recent.

The use of posttest mathematical adjustments that correct for misclassification have been proposed by several investigators [17]–[19], [21]. These adjustments rely on an accurate estimate of the anticipated false-recent rate in long-term infected participants [17]–[19], [21]. If the false-recent rate is accurate and relevant to the population being screened, the correction works quite well, as shown in the analysis of the VAX003 data (Table 2). However, relevant data may not be available, or special screening over time in the test population may be required to generate the data [30], [31].

Our results suggest that the BED estimate of incidence, when determined on specimens from prospective cohort studies of initially HIV-1–seronegative persons, is comparable to the independently estimated conventional incidence from the same cohorts in Thailand, both for CRF01_AE and subtype B′.

The cohort design allows one to identify, model, and quantify factors that perturb the estimate, two of which are noted here (unstable incidence and the significant impact of long-term prevalent specimens that may register false-recent in the assay). The availability of tests for determining incidence has multiple potential advantages, not the least of which is that an incidence testing program can easily be superimposed on surveillance programs for HIV-1 prevalence. Several countries, Thailand included, are supplementing national surveillance for HIV infection by using BED-based incidence estimation while incorporating elements of case-based surveillance. A recent survey of Thai military conscripts during 2005 and 2006 found a BED-estimated incidence of 0.14 to 0.20% per year [33], a significant decline compared with the estimate of 1.19 per 100 PY for 1991–1993 (Table 1).

Many of the survey design, data collection, and sampling issues related to prevalence estimates also apply to incidence estimates. However, there will be issues, expected or otherwise, that are unique to incidence testing and particularly to population-based versus cohort-based settings. In implementing the BED method for population-based surveillance, it will be important to be aware of the biases, assumptions, and limitations of making incidence estimates and to mitigate their impact by careful survey design, testing, analytic adjustment, and extrapolation.
